# An Algorithmic Approach for Detecting Bolides with the Geostationary Lightning Mapper

**DOI:** 10.3390/s19051008

**Published:** 2019-02-27

**Authors:** Clemens M. Rumpf, Randolph S. Longenbaugh, Christopher E. Henze, Joseph C. Chavez, Donovan L. Mathias

**Affiliations:** 1 NASA Advanced Supercomputing Division, NASA Ames Research Center, Moffett Field, CA 94035, USA; randolph.s.longenbaugh@nasa.gov (R.S.L.); chris.henze@nasa.gov (C.E.H.); donovan.mathias@nasa.gov (D.L.M.); 2 NASA Postdoctoral Program, USRA, Mountain View, CA 94043, USA; 3 Independent Researcher, Albuquerque, NM 87111, USA; chavezaurus@gmail.com

**Keywords:** meteor, asteroid, GLM, GOES, lightning, Geostationary Lightning Mapper, bolide, light curve, asteroid, Cuba

## Abstract

The Geostationary Lightning Mapper (GLM) instrument onboard the GOES 16 and 17 satellites can be used to detect bolides in the atmosphere. This capacity is unique because GLM provides semi-global, continuous coverage and releases its measurements publicly. Here, six filters are developed that are aggregated into an automatic algorithm to extract bolide signatures from the GLM level 2 data product. The filters exploit unique bolide characteristics to distinguish bolide signatures from lightning and other noise. Typical lightning and bolide signatures are introduced and the filter functions are presented. The filter performance is assessed on 144845 GLM L2 files (equivalent to 34 days-worth of data) and the algorithm selected 2252 filtered files (corresponding to a pass rate of 1.44%) with bolide-similar signatures. The challenge of identifying frequent but small, decimeter-sized bolide signatures is discussed as GLM reaches its resolution limit for these meteors. The effectiveness of the algorithm is demonstrated by its ability to extract confirmed and new bolide discoveries. We provide discovery numbers for November 2018 when seven likely bolides were discovered of which four are confirmed by secondary observations. The Cuban meteor on Feb 1st 2019 serves as an additional example to demonstrate the algorithms capability and the first light curve as well as correct ground track was available within 8.5 hours based on GLM data for this event. The combination of the automatic bolide extraction algorithm with GLM can provide a wealth of new measurements of bolides in Earth’s atmosphere to enhance the study of asteroids and meteors.

## 1. Introduction

A new space-based remote sensing system designed to detect terrestrial lightning activity called the Geostationary Lighting Mapper (GLM) aboard the GOES 16 and GOES 17 satellites [1], operated by the National Oceanic and Atmospheric Administration (NOAA), has recently become operational. Together, the GLM sensors on GOES 16 and GOES 17 cover about half of the Earth’s surface with overlapping viewing fields over the Americas. Although the GLM system is originally designed to capture natural lightning activity, it is well capable of detecting large meteors, called bolides [2,3]. In addition to its large coverage area, which potentially allows it to capture unprecedented numbers of meteors, its data is freely accessible to the public. 

Given that the system was originally designed to capture lightning activity in the atmosphere and produces about 1.2 GB of data each day, a new algorithm is required that can sieve through the large quantity of data to automatically extract meteor signatures. 

Here, we present an algorithm that uses the GLM Level 2 data product as input and filters through the data to extract bolide candidate signatures. Signatures are comprised of time-stamped ground track information as well as light curve intensity. Such data is valuable for future analysis such as orbit determination [4] and compositional investigation of the body [5]. In addition, meteor observations, such as delivered by GLM, may help to analyze the flow regimes [6] encountered by the meteoroid as it travels through the atmosphere and to reconstruct their entry trajectory. The latter application is particularly useful to the recovery meteorites for further analysis [7,8].

Section 1.1 provides a short introduction into meteors and places GLM in context to some of the systems that are in use today to detect them. Section 1.2 introduces the sensor itself while Section 1.3 outlines the size range of meteoroids detectable by GLM. The filters and the architecture of the automatic algorithm are described in Section 2. In addition, this section details the differences between lightning and bolide flashes. The algorithm’s performance and its sensitivities are discussed in Section 3. This section highlights bolide discoveries made with the algorithm as well as the challenge of extracting small bolides. The Cuban meteor case study is noteworthy as a performance demonstration and it is discussed in Section 3.5.

### 1.1. Meteors and Meteor Detection Systems

It has been estimated that more than 50 t of meteoroidal material collides with the Earth daily [5,9]. The asteroid size – impact frequency relationship indicates that most colliding objects are as small as dust grains and remain undetected [10,11]. However, 1 m-sized objects are expected to collide with the Earth every two weeks on average and these objects release sufficient kinetic energy when entering the atmosphere to trigger sensors that monitor transient occurrences in the atmosphere. In fact, the kinetic energy of these objects quickly reaches levels comparable to nuclear weapons because their impact speeds range between 11–72 km s^−1^ [12,13]. A recent example is the 2013 Chelyabinsk superbolide that registered at 550 kilotons Trinitrotoluene (kt TNT) equivalent (comparable to about 36 times the energy yield of the Hiroshima bomb) when it disintegrated over northern Russia. This superbolide, estimated at about 18 m in diameter, injured over 1500 people [14,15]. 

Most of the large bolides are thus recorded by a network of ground-based sensors that is intended to enforce the nuclear test ban treaty (operated by the Comprehensive Nuclear-Test-Ban Treaty Organization, CTBTO) and is capable of detecting such occurrences globally [16,17,18]. The sensor network is designed for national security applications and therefore only publishes a significantly reduced data set it deems safe to present to the public. Published detections include the geolocated position and time of peak brightness, as well as cumulative energy release, but no light curve or ground track information.

Of similarly confidential nature as the CTBTO sensor network are space-borne, military United States Government (USG) sensors [3]. Occasionally, researchers may gain access to portions of the collected data by these sensors but the data products are generally not available to the public.

Local ground camera systems that usually use fisheye lenses to record the entire, locally visible sky (e.g. European Fireball Network, Desert Fireball Network, NASA All Sky Fireball Network, Sky Sentinel, Cameras for Allsky Meteor Surveillance, Spanish fireball network, Fireball Recovery and Interplanetary Observation Network - FRIPON) can detect smaller meteors and provide open data access [19,20,21,22]. That data regularly includes flight path and lightcurve information. However, these systems only have a limited detection range around their installation sites on the Earth. The total sky coverage of these systems remains sparse compared to Earth-viewing satellite systems. In addition, most ground based camera systems can easily saturate for larger events and do not produce accurate disintegration recordings.

With GLM, the scientific community gains access to a system that is open to the public, has semi-global coverage, and provides ground track as well as light curve information. GLM will provide significant value to the meteor detection community when it is used in conjunction with the systems mentioned above. The algorithm presented here, enables access to this data which would otherwise by treated as noise.

### 1.2. Geostationary Lightning Mapper Instrument

**Figure 1:**
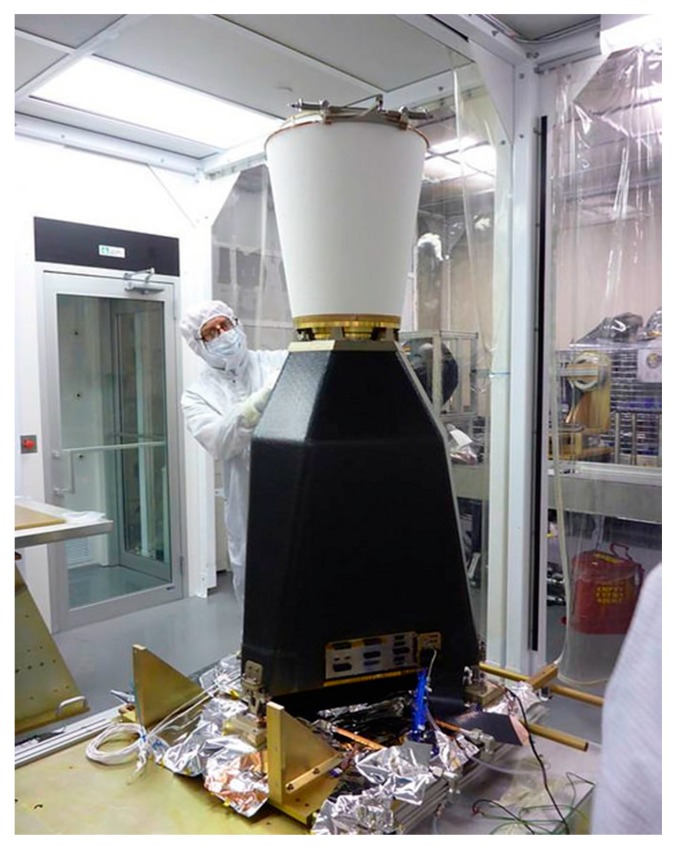
GLM assembly with sun shade pre-launch [23].

The GOES Geostationary Lightning Mapper [1] records from geostationary orbit transient light events at a rate of 500 frames per second (2 ms). GLM has a staring CCD imager (1372 × 1300 pixels) with a narrow 1.1 nm pass-band centered at 777.4 nm, a wavelength associated with the neutral atomic oxygen emission (OI) line of the lightning spectrum. Figure 1 shows the GLM system being assembled at the Lockheed Martin Advanced Technology Center in Palo Alto, CA with a worker in the picture for scale. 

Although GLM has been designed to detect lightning, it detects bolides as well. For a detailed analysis of how GLM responds to energy emitted by bolides the reader is referred to [2].

**Figure 2:**
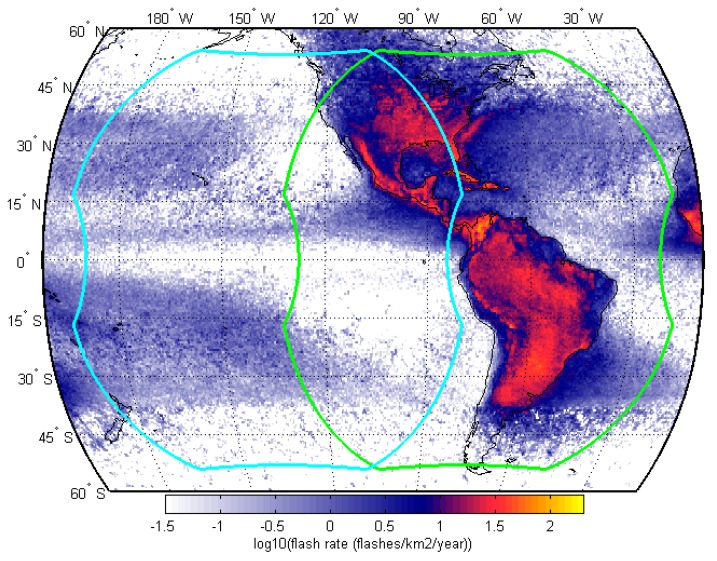
GLM coverage showing both GOES-16 (green) and GOES-17 (cyan) on map of lightning flash rate [24].

GLM provides continuous coverage from geostationary orbit and covers approximately half of the Earth with the Americas as the primary land-mass focus. Its spatial resolution is in the range of 8–14 km. Currently, two GLM sensors are in space: GOES 16 occupies the geostationary orbital slot at 75.2 degree west and GOES 17 is located at 137.2 degree west. Figure 2 shows the geographical coverage for both GLM sensors over a map of lightning intensity. Parts of the United States and the Pacific have overlapping fields of view of the two GLM sensors potentially enabling stereo bolide detections.

### 1.3. GLM’s Meteoroid Detection Size Range

Brightness determines GLM’s meteor detection range. Towards the small end of the detection range, GLM is limited by its minimum required brightness level and it has been reported in [2] that the detection threshold is about −14 visual magnitude. At visual magnitude −14, comparable to twice as bright as the full moon, a meteor is considered a bolide [25]. It is expected that a meteor needs to be at least decimeter-sized (in diameter) to reach bolide brightness levels and, thus, to be detectable by GLM [5]. On the other hand, GLM’s sensor will saturate for objects measuring multiple meters in diameter due to their excessive energy release [2]. A saturated sensor will reduce data quality of light curves and ground tracks. As such, GLM’s target range will be for objects ranging from decimeter to meter size in diameter. 

**Figure 3:**
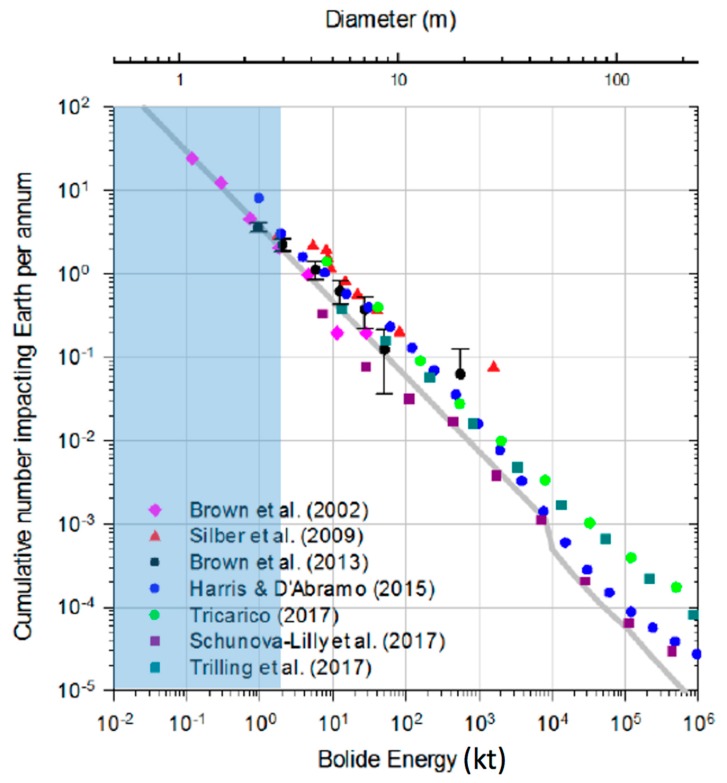
Impact frequency over mean impact energy (and estimated diameter). The grey line represents a fit through actually recorded bolide events presented in several publications (see legend) based on several sensor systems. The light blue shaded area is the expected detection range of GLM and it complements the available data towards smaller sizes [27].

Figure 3 presents size and impact frequency for bolides. It is estimated that a 1 m diameter small asteroid collides with the Earth every two weeks, while a 4 m diameter object hits the Earth roughly once per year [26]. The shaded blue area in Figure 3 approximates the usable bolide detection regime expected of GLM. It extends from 0.1 m (outside the figure) to 3 m. The axis conversion of impact energy and object diameter fits the assumption of a meteoroid density of 2300 kg m^−3^, and an impact speed of 21 km s^−1^. The data points in the figure correspond to other sensor systems such as CTBTO and space based USG sensors. The expected GLM detection regime complements the detection range provided by these remote sensing systems towards smaller sized meteors. 

## 2. Bolide Extraction Algorithm

This section highlights data signatures produced by GLM and presents the bolide extraction algorithm which takes advantage of the differences between bolide and lightning signatures to identify those signatures that are bolide-like. Six filters have been devised to extract bolides signatures and they are presented in the following. 

### 2.1. Definition of Terms

Transient optical events are detected by the GLM. The data that is produced on-board is labeled as the Level 0 (L0) data product. This data is subsequently sent to the ground where it is post-processed into the Level 2 (L2) data product, which is published and available to the public. It is worth mentioning that the algorithm presented here relies purely on the publicly available L2 data product. The processing from L0 to L2 aims to decrease the amount of noise in the data and clusters the optical events into logical entities such as groups and flashes based on spatial and temporal threshold parameters as described in the Product Definition and Users’ Guide Volume 5 [1,28,29]. 

**Figure 4:**
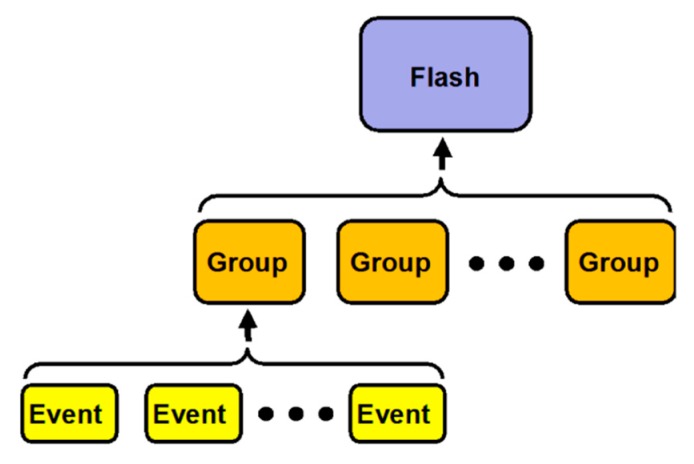
Hierarchical relationship between a flash, groups and events [28].

There are three data structures in the GLM level 2 relevant to this document. They are events, groups and flashes. Although conceived for applications in a lightning context, these data structures are not lightning specific and can be understood as a general GLM data concept. An event represents a light emission signal that stood out against the background image seen by the GLM sensor. An event occurs in an individual pixel over a 2 ms integration period. A group represents the collection of events detected in adjacent sensor pixels over the 2 ms integration period. A group’s energy is equal to the sum of its event’s energies and the location of a group is the energy weighted average location of its events. A flash represents a series of measurements constrained by temporal and spatial extent thresholds that are associated with one or more groups. In a hierarchical sense, a flash is at the top, followed by a group, with events representing the smallest entity as visualized in Figure 4. The algorithm presented here mainly utilizes the group structure.

### 2.2. Collecting GLM Groups Into Flashes

The GLM L2 data can be read using a netCDF4 file reader. Even though the GLM L2 data product already provides groups that were clustered into flashes, we employed an in-house clustering algorithm to maintain flexibility in case the original, lightning-focused clustering would prove detrimental to group clustering for bolides. The literature provides a thorough description of the original GLM clustering algorithm [1,29]. In a first step, the algorithm sifts through the data and collects groups, that are spatially and temporally close, into flashes. The algorithm compares each group to its following group and assesses if its location and timestamp falls within the threshold values listed in Table 1. All groups for which the threshold is met successively without interruption are collected into one flash. These threshold values have been selected early in this analysis on a trial basis and were sufficient for the purpose of bolide detection. 

### 2.3. Flashes

After collecting groups into flashes, the flash objects are assessed for their resemblance to expected bolide behavior. This section presents a recorded lightning and a real bolide flash emphasizing distinguishing features to inform the subsequent filtering process.

#### 2.3.1. Lightning Behavior

**Figure 5:**
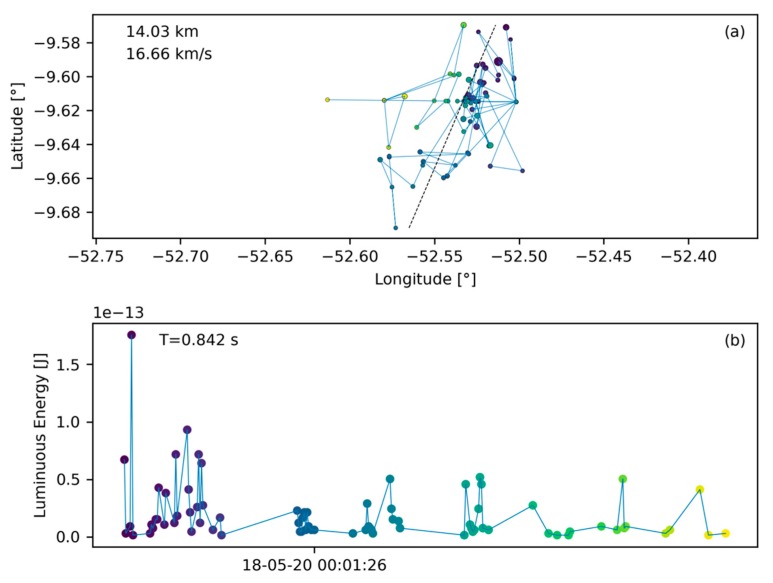
A lighting flash with its ground path (made up of groups) in plot (**a**) on a latitude/longitude grid and its light curve in plot (**b**). The light curve provides the luminous energy at the baffle of the GLM instrument (before passing the lens) in Joules. The abscissa-axis provides the date and time (format: YY-MM-DD HH:MM:SS in UT) of the signature and the annotation in the top left of plot (b) yields the signature duration. The annotation in in the top left of plot (a) shows the largest distance between two points of the ground track and the speed is calculated based on the division of that distance and the signature duration. The color coding of the dots help to correlate ground track points with light curve data (dark points indicate earlier groups while yellow points indicate the end of the signature). In addition, the size of the ground track points is proportional to the energy value of the corresponding light curve point, meaning that larger points correspond to a larger amount of recorded energy.

The most common data entity found in the GLM L2 data are lightning flashes as shown in Figure 5. This figure presents the ground track of the flash in plot (**a**) on a latitude/longitude coordinate grid (in degrees). Each ground track point is a group location as assigned in the GLM L2 data product. The distance between the two furthest ground track points is annotated in the top left corner conveying a sense of scale. Below the distance, speed is annotated which is calculated as the division of the ground track extent by the signature duration. Note that the ground track only shows the horizontal component of the trajectory (more relevant for meteors), equivalent to a projection of the trajectory onto the ground. Speed estimates are therefefore only indicative and may differ significantly from the true speed. For illustration, consider a meteor that enters on a near vertical trajectory in which case the ground track extent will be small and the speed estimate is low. Hence, the speed tag only shows the horizontal component of the meteor velocity. The signature duration is noted in the top left of plot (b). Plot (b) presents the light curve in terms of received luminous energy at the aperture of the GLM in Joules over time. This is not the total energy emitted by the bolide and further scaling is necessary for such an estimate [2,3,26,30]. For formatting reasons only the date and time stamp corresponding to the signature’s middle group is shown. Together with the signature duration, these coordinates provide adequate information about the flash occurrence time and its persistence. 

Common lightning flashes are characterized by a seemingly random ground path (Figure 5a). In contrast, bolides are expected to travel along straight lines. Hence, a first step for the algorithm presented here is to perform a line fit to the ground path data and this fit is represented by a thin, black, dashed line in Figure 5a. The average residual of such a fit (average residual $R=4.425\times 10^{-4}$ in this case) will be used to assess if a flash could be a bolide or not (Section 2.4.2). We assume further, based on our experience having seen numerous lightning profiles in L2 data, that a typical lightning flash deposits its energy randomly over time without a temporal preference for peak energy deposition as demonstrated in Figure 5b. Particularly, there is no predominant and extended energy deposition feature which would be characteristic for a bolide. 

#### 2.3.2. Confirmed Bolide

**Figure 6:**
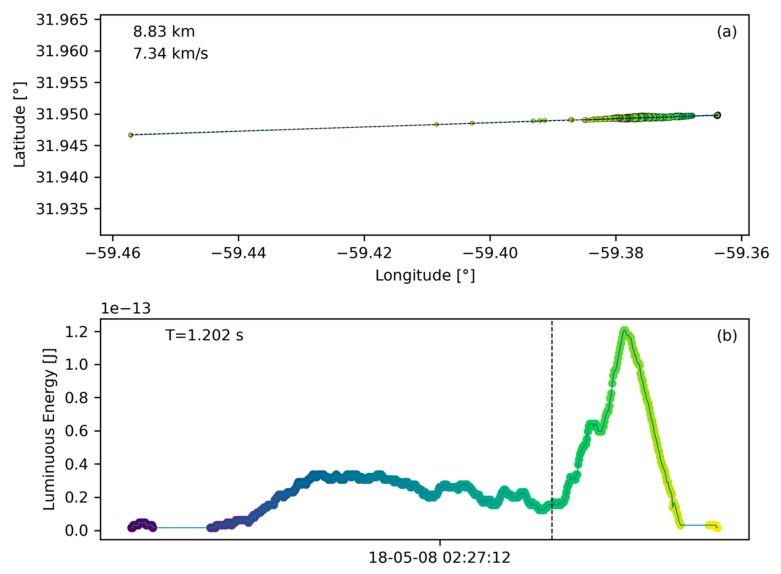
A bolide recording (true positive) confirmed by USG sensors. The ground track in plot (**a**) can be well approximated by a straight line. Plot (**b**) shows the light curve over time. The dashed energy balance line indicates that half of the energy was deposited only in the last 27% of total bolide duration.

To illustrate the appearance of a typical bolide signature, a bolide that occurred at 02:27:12 UT on May 8th 2018 over the Atlantic Ocean and was confirmed by USG sensors [31] is shown in Figure 6. Plot (**a**) contains the ground track and plot (**b**) shows the light curve. Bolide confirmations in this manuscript rely on detections of the same flash from multiple sources and the corraborating source is mentioned or cited in all presented cases. The typical confirmation process involved checking if other systems (such as data published on the JPL fireball website [31], or ground based camera systems) had a detection at the same location and time as identified by GLM. In some cases, such as shown in Figure 6, we were able to perform a more comprehensive comparison of GLM detections with USG sensor data which was available to us. The bolide’s signature contrasts to the lightning data shown in Figure 5. The ground path is significantly more line-like (average residual $R=4.938\times 10^{-9}$) than the lightning flash example and the dashed fit line in Figure 6a is congruent with the actual bolide ground path. Furthermore, as the meteoroid enters the atmosphere and begins to ablate and fragment, a limited amount of its kinetic energy is deposited in the early phase of the signature. Subsequently, the fragmentation cascades into an explosion-like energy deposition which is represented by a clear peak in the last third of the energy deposition timeline shown in Figure 6b. Compared to Figure 5b, the light curve appears smoother and with a more defined and pronounced peak than the lightning flash. For illustration purposes, a vertical dashed line in Figure 6b indicates the point in time when half of the total energy of that bolide is emmitted and here that value is after 73% of the signature time has passed. It is expected that bolides show a clear bias towards late energy deposition in contrast to lightning which is not expected to have a general bias. 

### 2.4. Individual Filter Design

This section describes the design of the six filters that comprise the bolide extraction algorithm. Two of the six selected filters work on characteristics that are inherent in ground track information. They are the “Line Fit Filter” (Section 2.4.2) and the “Group to Line Distance Filter” (Section 2.4.4). Another two filters work on light curve characteristics and those include the “Energy Balance Filter” (Section 2.4.3) as well as the “Polynomial to Energy Moving Window Filter” (Section 2.4.5). The last two filters utilize metadata for the “Group Count Filter” (Section 2.4.1) and the “Signature Duration Filter” (Section 2.4.6). The individual filters are combined to form the extraction algorithm and the corresponding architecture is described in Section 2.5. 

The six presented filters are the result of an extensive trial and error process that included adjustments to the filter metrics and exploring filter concepts that proved impractical or ineffective. One example of an unused filter concept includes extracting patterns from latitude and longitude time histories before settling on the combination of the two in the form of ground track plots. Another example of positive filter development is the switch from raw data to normalized light curve and ground track data to increase the robustness of some filters against variations in bolide flash magnitudes.

The filters operate on specific signature metrics and sigmoid functions were used to transform individual metric values into bolide scores ranging from zero to one. A larger score value reflects better agreement with behavior that is expected for bolides. The family of sigmoid functions is defined with:

(1) $$S\left(x\right)=\frac{1}{1+e^{-a\left(x-b\right)}}$$

where S denotes the individual filter score, x is the filter metric or free variable, a determines the steepness of the transition region of the function, and b assigns the free variable value for which $S_{i}\left(x=b\right)=0.5$. In the cases of the line fit residual filter, the line distance ratio filter, the polynomial fit filter, and the signature duration filter, the sigmoid function needed to provide smaller values for larger input values. In those cases, the structure of the sigmoid function was changed to:

(2) $$S\left(x\right)=1-\frac{1}{1+e^{-a\left(x-b\right)}}$$

**Figure 7:**
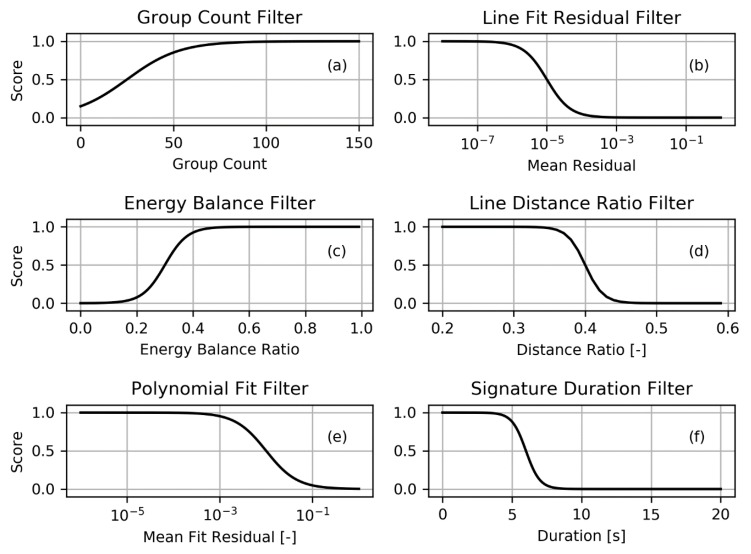
Filter functions providing the passing score in the range [0,1] for each test. A larger score is returned when: Plot (**a**)—more groups constitute a flash; Plot (**b**)—the line fit returns a low residual; Plot (**c**)—the flash energy balance is skewed to later times; Plot (**d**)—the maximum observed distance between any group and the fitted line is small; Plot (**e**)—the polynomial fits return smaller residuals; Plot (**f**)—the signature’s temporal duration is shorter.

Figure 7 visualizes the six filters used in the algorithm presented here. The following sections detail the derivation of each filter and the baseline parameters are listed in Table 2. 

#### 2.4.1. Group Count Filter

**Figure 8:**
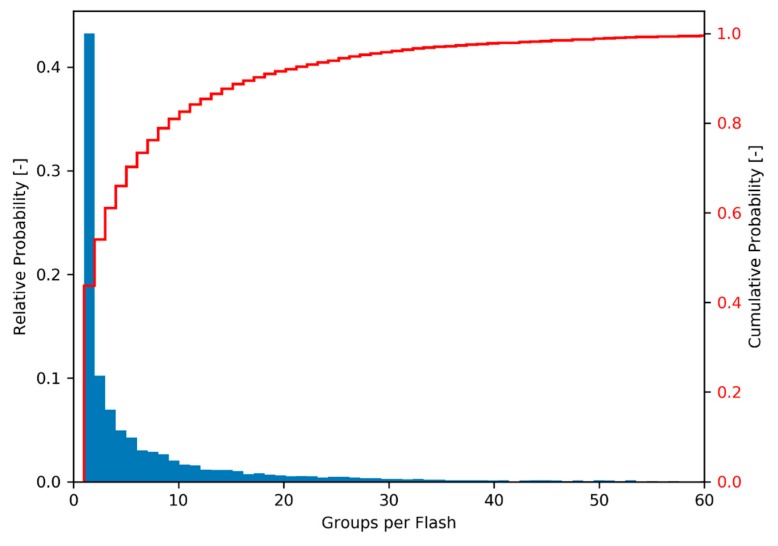
Histogram showing the occurrence rate of flashes with a given number of groups. The random sample of GLM data shows 8864 flashes from 3 minutes and 20 seconds worth of data. Over 91% of the flashes in the sample consist of less than 20 groups.

The smallest measurement entity that is utilized by the algorithm in Level 2 data is the GLM group (Section 2.1). With this basis, the algorithm searches the data sequentially for other groups that are spatially and temporally close (Table 1) and collects those into flashes which then can become bolide candidates after passing the filters. The first filter for a bolide flash to pass in order to be considered a bolide candidate is the minimum number of groups that constitute a bolide flash. Individual light emissions in typical lightning storms, are short and distinct, triggering only a limited number of group recordings, resulting in a low group count (compare number of data points in Figure 5b with Figure 6b). Conversely, bolides emit light continuously and over longer periods than a number of light emissions in a lightning storm. It is expected that bolide flashes in GLM data distinguish themselves from lightning data by a larger group count per flash. To support this observation, the histogram in Figure 8 shows the distribution of group count per flash based on three minutes and twenty seconds worth of randomly selected data. Only 9% of the recorded flashes have more than 20 groups. For comparison, the bolide portrayed in Figure 6 corresponds to a flash with 519 groups. In fact, flashes with large group counts are outliers and represent an effective filtering opportunity for bolides. It is therefore prudent to filter out flashes with a small number of groups as those are likely lightning and to pass those with large group counts. Here, the b-parameter of the group count filter was set to b=25. Based on extensive algorithm sensitivity analysis baseline filter parameters were chosen and they are listed in Table 2.

#### 2.4.2. Line Fit Filter

**Figure 9:**
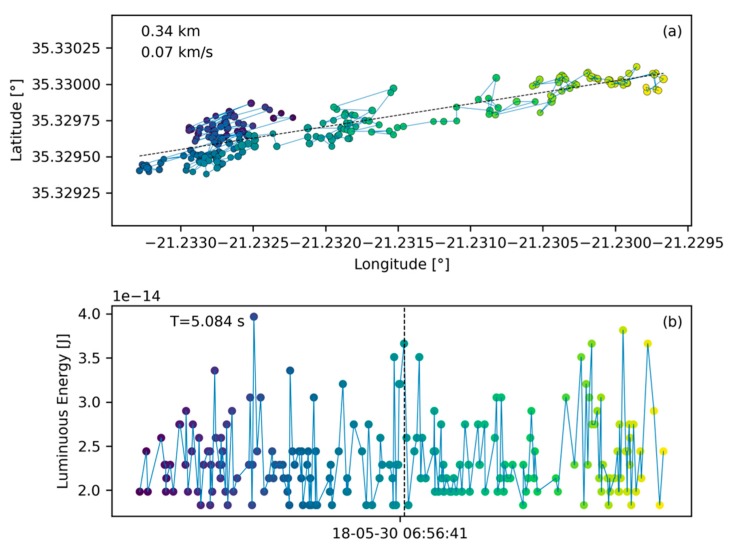
A false positive detected by the algorithm. Ground track is shown in plot (**a**) together with a least-squares fit line. The ground track exhibits considerable jitter relative to the line approximation. Plot (**b**) shows the energy deposition profile over time.

Meteors enter the atmosphere with a speed of at least 11 km s^−1^ and remain on an approximately straight trajectory along their short visible arcs as they burn and ablate. Hence, a line is well suited to approximate the ground path of the flash. In contrast, a flash caused by lighting, exhibits a substantially higher degree of randomness in its ground track. Figure 9 provides an example for such random walk in plot (a). Even though the overall motion has a tendency to follow the fitted dashed line, individual track points may move in every direction away from or across the line. Figure 5a showcases this randomness yielding no directional trend in this example. In comparison, the bolide ground track of Figure 6a follows the expected straight path. This clear distinction in the ground paths of lighting and bolides facilitates filtering through line fitting. Specifically, a line is fitted to the ground track of a flash and the residual is evaluated subsequently to assess the quality of the fit. In Equation (3), the average line residual ($R_{L}$) is defined as the sum of squared deviations ($d_{j}^{2}$) of the group points (j) from the fitted line, divided by the total number of groups (N):

(3) $$R_{L}=\frac{\sum_{j}^{N}d_{j}^{2}}{N}$$

Because a lower residual indicates a closer fit and should therefore receive a larger filter score, the parent sigmoid function of Equation (1) was changed to reflect this behavior and the adapted sigmoid function of Equation (2) was used instead. Furthermore, because the range of residuals was large, the base 10 logarithmic value of the residual value was chosen as the filter parameter $x=\log_{10}R_{L}$. With these adaptations, the line filter function is plotted in Figure 7b using the parameters listed in Table 2. 

#### 2.4.3. Energy Balance Filter

The process of a meteor burning up in the atmosphere begins with a single body ablating at high altitude in low density atmosphere. These conditions yield a low energy deposition rate. As the meteor descends, fragments break off and contribute to the ablative energy deposition. In addition, increasing atmospheric density results in larger heating which promotes fragmentation. This process is self-reinforcing and leads to one or more explosion like flares. Figure 6b shows an example of such an energy deposition profile. The vertical dashed line in that figure annotates the time of half energy deposition for that profile. For bolides, the pre-half-energy duration tends to be long accounting for over 50% (or a fraction of >0.5) of total profile duration. As an example, for the case in Figure 6b this fraction is 0.72 because the pronounced flare peak emits the remaining half of the energy in the last 28% of the signature time.

In contrast, lighting emits energy sporadically without an apparent temporal bias towards the early or late profile duration. In fact, the energy half time in the lighting example shown in Figure 9b was reached after exactly half of the profile duration had passed. In this case, the half energy deposition fraction is 0.5 as equal amounts of energy are released before and after half of the profile period has passed. While the fraction might vary for individual lightning cases, it is expected that the mean of lightning energy deposition fractions is 0.5. Figure 7c visualizes the energy balance filter function which uses Equation (1) with the parameters of Table 2.

#### 2.4.4. Group to Line Distance Filter

While the residual of the line fit is computationally cheap early in the filtering process to discard unsuitable signatures, it allows for a large number of false positives to pass. The reason is that the line fit filter relies on the average discrepancy of all group points from the fit line. As a result, signatures that only have few points far away from the line pass the filter because their overall average is low. In other words, the line fit residual provides an aggregate value for the entirety of the ground track and does not check the fit quality of each individual point. Rather than considering the aggregate fit quality, the maximum distance filter described in this section considers each point in the ground track and calculates its distance to the previously obtained best fit line. Because the ground track follows a well predictable straight line for bolides, there is high confidence in the observation that each point should be close to the center line and no single point is allowed to deviate far from it. Therefore, this filter is a stricter version of the line fit filter which comes at the cost of larger computational expense. The larger computational cost is acceptable at this point because the set of remaining flashes is small compared to the starting set as earlier filters have discarded most flashes.

The angular distance in km is calculated between each group and the best fit line and the distance value of the group with maximum separation from the line is selected. This value is normalized to increase robustness against variations in bolide magnitude. The normalization is accomplished through division of the angular distance value by the overall angular span of the ground track. For the overall angular span, we use simply the larger value of the overall latitude or longitude span. The normalized distance allows for larger point-line distances for large bolide events as the overall lat/lon range increases. Equation (4) formalizes the distance ratio $T_{j}$ for group j where d_j is the angular distance between the group and the line, $\lambda_{max}$ denotes maximum longitudinal span of the ground track, and $\Phi_{max}$ denotes maximum latitudinal span of the ground track:

(4) $$T_{j}=\{\begin{array}{ll} \frac{d_{j}}{\lambda_{max}}, & \lambda_{max}\geq \Phi_{max}\\ \frac{d_{j}}{\Phi_{max}}, & \lambda_{max}<\Phi_{max} \end{array}$$

Given that smaller point-line distance values should correspond to larger filter scores, the sigmoid function structure of Equation (2) is used where $x=\underset{j}{\max}\left(T_{j}\right)$. The filter function is plotted in Figure 7d using the parameters shown in Table 2.

#### 2.4.5. Polynomial to Energy Moving Window Filter

**Figure 10:**
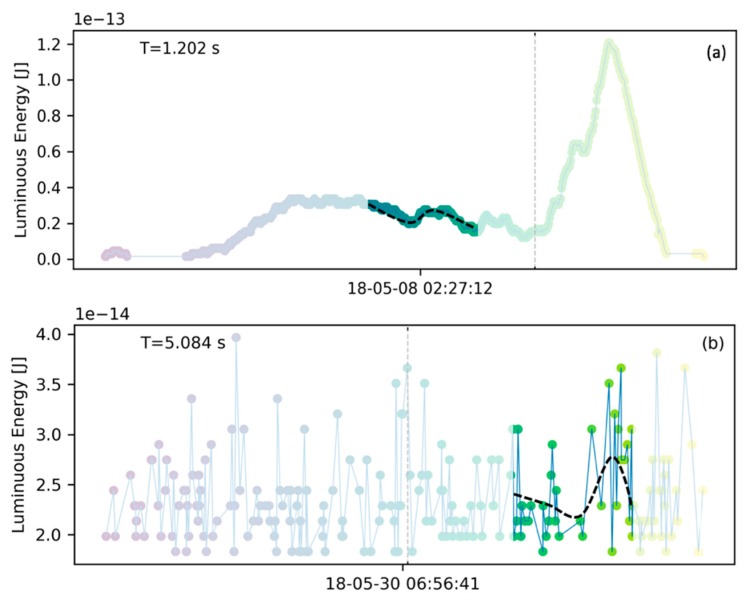
Visualization of the polynomial fit filter. Plot (**a**) shows a bolide light curve. The moving window span is indicated by the clear portion of the plot. A polynomial represented by a dashed line is notionally fitted to the light curve data. Plot (**b**) shows a non-bolide light curve and the fitted polynomial represented by the dashed line. The polynomial fits better the data of the bolide (a) than for the non-bolide (b).

The light curve of a bolide is generally smoother than that of lightning. This difference is exploited using a filter that fits a 3rd order polynomial to a small subrange of the light curve. Specifically, the subrange is defined here to be five groups wide. Figure 10 compares the light curve of a bolide in plot (**a**) with a lightning light curve in plot (**b**). 

A polynomial is notionally fitted to the highlighted subrange of the light curve data as a dashed black line. It is clear that in the case of the bolide (Figure 10a), the polynomial yields a closer fit than a similar fit to the lightning lightcurve (Figure 10b). The metric of the fit is the least-squares residual for the polynomial normalized by the energy range of the signature. Thus, the normalized polynomial residual $R_{P}$ is the sum of squared residuals $\sum_{i=k}^{k+5}d_{i}^{2}$ for the current subrange of the lightcurve divided by the squared energy range of the signature ${\left(E_{max}-E_{min}\right)}^{2}$ as shown in Equation (5). The residual normalization with the energy range is necessary because flashes with large energy releases (such as bolides) would otherwise yield large residuals even though the fit appears adequate:

(5) $$R_{P}=\frac{\sum_{i=k}^{k+5}d_{i}^{2}}{{\left(E_{max}-E_{min}\right)}^{2}}$$

The five group-wide lightcurve subrange moves across the entire signature and the polynomial fit is computed for each location. The input for the filter function visualized in Figure 7e is finally formed as the mean normalized residual of all subrange fits. The base ten logarithmic value of that mean serves as input to the filter function (Equation (2)) such that $x=\log_{10}\left[\underset{m}{\mathrm{mean}}R_{P_{m}}\right]$ if there are m subfits in a signature. Figure 7e visualizes the corresponding filter function based on the parameters listed in Table 2.

#### 2.4.6. Signature Duration Filter

A substantial amount of artifacts that appear in L2 data are reflections of the Sun on the surface of the Earth (glints). These tend to endure for longer than bolides could possibly last. Some instances of glints lasted for longer than 30 s. For comparison, the large Chelyabinsk super-bolide lasted for about 13s. To discard long duration artifacts, a signature duration filter has been implemented which is visualized in Figure 7f based on Equation (2) and the parameters listed in Table 2. In its baseline setting, it filters out signatures that last for longer than approximately 6 s. Even though bolides may last longer than 6 s, this value was deemed appropriate because very large bolides are rare and would be noticed by other systems with global coverage (such as USG sensors) and could be extracted from GLM data manually. 

### 2.5. Algorithm Architecture

**Figure 11:**
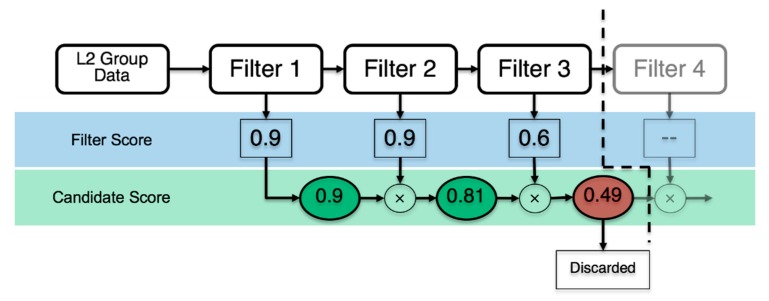
Algorithm architecture. The L2 group data passes through each filter sequentially and the product of the individual filter scores forms the cumulative candidate score. If the cumulative candidate score falls below the threshold value (here 0.5), the signature is discarded.

Our initial algorithm architecture was driven by sequential filtering steps with distinct threshold values. Flash signatures had to pass all to be classified as bolide candidates. This approach did not prove adequate for the diversity of signatures present in the GLM L2 data. Tuning the thresholds in such a way that they would consistently allow true positives to pass while filtering out obvious lightning and other artifact signatures was impractical and resulted in either excessively restrictive (not passing true positive signatures) or too relaxed filters (passing too many false positives). With this insight, we improved the procedure by replacing the distinct threshold values with an overall score aggregated from the individual filter scores in the range [0,1]. Continuous sigmoid functions (Figure 7) represent each filter and they assign a value between zero and one based on the signature’s similarity with bolide characteristics. Following each filter step, the cumulative bolide score is calculated through multiplication of all previous filter scores. Hence, the overall bolide score for a signature after passing six filters is given with:

(6) $$\Theta =\prod_{i=1}^{6}S_{i}$$

where Θ is the cumulative filter score, and $S_{i}$ denotes the individual filter score by the $i^{th}$ filter. The overall filter score Θ needs to pass a minimum threshold score to be considered a bolide candidate. This cumulative threshold score is a tuning parameter of the filter and a value of 0.5 was found to be a suitable first guess. Figure 11 illustrates this algorithm architecture. 

Typically, a real bolide will score close to one in all filters and, thus, receive a cumulative bolide score close to one. However, using the score-based approach also allows for a signature with deviations from an ideal bolide signature to be considered as bolide candidate when it receives a relatively low score in one or two of the filter metrics. As long, as the cumulative score remains above the threshold for the cumulative score the flash can still be considered a candidate and the algorithm is thus amenable to natural variations in bolide signature appearances. 

On the other hand, an unwanted lightning flash might resemble bolide signatures in few of the metrics but it is unlikely that it scores sufficiently high in enough metrics to pass the cumulative score threshold. By forming the cumulative bolide score through multiplication of individual filter scores an overall low score for lightning flash signatures is enforced and the signature is discarded.

## 3. Filter Sensitivity and Algorithm Performance Discussion

### 3.1. Filtering Rate and Sensitivity Sample

The bolide extraction algorithm has been analyzed for its overall performance. About 34 days- worth of data or 144845 files (each accounting for 20 s worth of data) were processed with the algorithm using the baseline parameter set shown in Table 2. The yield was 2252 files which contained bolide candidate signatures. This performance corresponds to a pass rate of 1.55% of analyzed files. The algorithm has been implemented in Python and typically runs on a desktop computer using seven processing cores. With such an arrangement one day worth of data can be processed within 2-3 hours. As this is a prototype, there are multiple avenues for processing speed increases such as an implementation in a faster language (for example C++) or to run it on an increased number of processing cores. Memory usage is minimal and is not a challenge for a common desktop PC. To understand algorithm sensitivities, an additional downselection was performed yielding the evaluation subset. The evaluation subset was generated in order to facilitate short runtimes for each sensitivity iteration. To obtain a clear feedback signal about the performance of the algorithm given a combination of filter parameters, the subset has an increased ratio of passing to rejected signatures relative to a generic set of files. The larger number of passing signatures allowed us to assess how a change in filter parameters affected the number of passed signatures as well as the characteristics of signatures that were filtered out. Facilitated by the Hyperwall facility at NASA Ames Research Center (ARC) (the Hyperwall facility uses a configuration of 8 x 16 screens to visualize large quantities of data), the 150 file counting evaluation subset was derived by manually selecting 122 files with highly bolide-similar signatures from the filtered set and adding 6 confirmed (confirmation was based on USG sensors and/or meteor detection camera networks) bolide signatures, as well as 22 random data files. The evaluation subset was used to assess the effectiveness of each individual filter. 

### 3.2. Filter Performance

**Figure 12:**
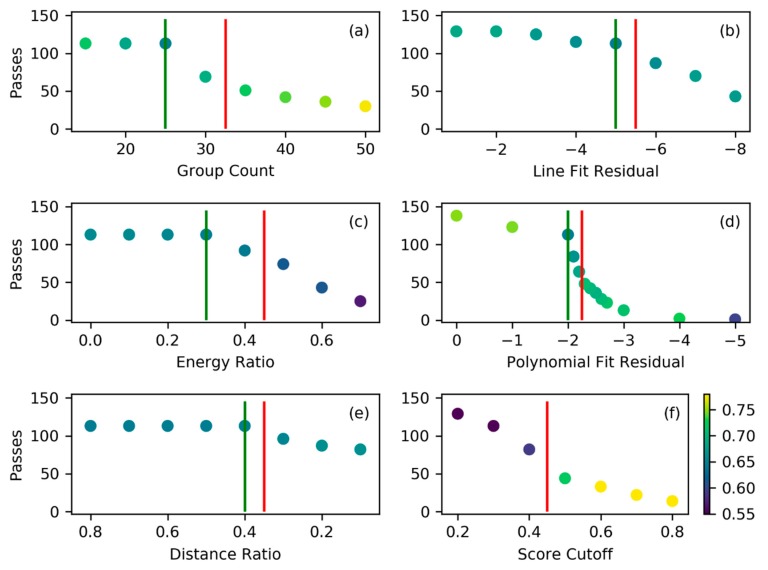
Filter effect on number of bolide candidate passes. The plots are oriented such that the filter parameters further to the right correspond to a more restrictive filter setting. The green line indicates the baseline filter setting. The red line marks the filter parameter step when a known true positive bolide signature was filtered out from the evaluation subset corresponding to an overly restrictive filter setting. The color shading of the points corresponds to the mean bolide score in the passed instances (colorbar shows score values). The shown filters are group count (**a**), line fit (**b**), energy ratio (**c**), polynomial fit (**d**), distance ratio (**e**), and cumulative bolide score cutoff (**f**).

The filter effectiveness was tested by varying one filter parameter at a time while keeping the other parameters at the baseline setting (Table 2). Figure 12 presents the outcome of the performance analysis. The tested filter values are marked on the x-axis and they correspond to the b-parameter shown in Table 2, while the y-axis denotes how many signatures of the evaluation subset were passed with this particular setting. 

The filter performance results in Figure 12 demonstrate that all filters are effective in downsampling signatures when assigning more restrictive filter parameters (going left to right in the plots). This is evident by the fact that the number of passes decreases for more restrictive filter settings. It is also important to note that each filter (except for the distance ratio filter) begins to be effective in filtering out unwanted signatures before filtering out a known true positive bolide signature (indicated by the red line). This indicates that the filters work as intended and have the positive effect of discarding unwanted signatures and allowing bolide-similar signatures to pass. The main reason why the distance ratio filter seems to be ineffective before it discards confirmed bolide cases is due to the initial sample selection. The evaluation sample had been vetted manually prior to the sensitivity analysis and candidates which are obviously not bolides were already discarded. Signatures with large outliers are easily identified as non-bolides and therefore did not make it into the evaluation sample. Furthermore, it is likely that the line fit already removed those signatures in the 22 random files in the evaluation set which would otherwise be discarded by the distance filter. Hence, the distance filter appears ineffective for the evaluation subset even though it performs as intended. In other words, the evaluation subset is a challenging set for the filter algorithm as the majority of its content is already made up of bolide-like signatures rather than random signatures that are clearly not bolide-like. It is harder for the filter algorithm to discard the preselected candidate signatures because they are similar to bolides. 

Figure 12a shows the performance of the group count filter as the minimum number of groups per flash is increased (going left to right in the plot). The color shading of the points indicates the mean bolide score of the set that passes the filter. In the first three steps, this filter reduces the mean score of the passing set before the lowest scoring signatures are filtered out. Removing these low scorers raises the mean score of the remaining set. This trend continues to more restrictive filter values. This desirable behavior is also visible in Figure 12b, Figure 12d, Figure 12e, and Figure 12f representing the line fit, polynomial fit, distance ratio, and cutoff score parameters, respectively. 

The only filter that does not increase the mean score of the passed files for an increasingly restrictive filter setting is the energy ratio filter shown in Figure 12c. This observation indicates that, by tightening the filter, all signatures receive a lower bolide score rather than just the unwanted signatures. While still being an effective filter in general, it does not increase data quality as efficiently as the other filters. The reason for this behavior is that the assumption of a clear energy deposition bias in the light curve breaks down for very small bolides and this phenomenon is discussed further in Section 3.3.

The polynomial fit filter is noteworthy because it shows the steepest transition region of all filters in the parameter range that reduces signature passage rate suggesting that it is highly effective. However, the point of overly restrictive filter setting is located in the steep transition region indicating that the filter is easy to overtune. 

All filters show a plateau-like behavior initially and this is connected to the biased evaluation subset. The majority of the files in the evaluation subset (122) were selected after they had already passed the baseline filter setting. Therefore, these files will not be filtered out until the individual filter becomes as restrictive as the baseline setting. Those files that are filtered out in the plateau region are the random data files which have not necessarily passed the baseline filter previously. The distance ratio filter shown in Figure 12e visualizes this concept clearly as no files are filtered out before the filter passes the baseline setting.

The effect of raising the overall bolide score cutoff value is shown in Figure 12f. Increasing the cutoff value continuously reduces the pass count but, unsurprisingly, has the effect of increasing the mean bolide score of the set that passes the algorithm. It should be noted that for this test, the filter settings were tightened (for example polynomial = 2.2 and group count = 30) such that the pass count at the baseline setting (0.5) is lower than in the other filter plots. Finally, due to its late addition to the algorithm after the presented test, the signature duration filter is not present in Figure 12. 

The baseline parameter set (Table 2) represents a suitable filter parameter selection for effective bolide extraction algorithm operations and this setting is used today at NASA ARC to assess GLM data for bolides on a daily basis. All parameters (green lines) are on the relaxed side of the red lines in Figure 12 that indicate the borders to an overly restrictive filter setting. It should be noted that it would be impractical to set all parameters equal to, or very close to, the red line setting as this would result in an overly restrictive filter algorithm. The individual filters are connected to each other through the multiplication that yields the overall bolide score. Hence, if all filters are borderline restrictive, the overall bolide score will certainly drop below the score threshold and good signatures could potentially be discarded. This is especially true for small bolide events that only produce a weak bolide signature which we discuss in the next section.

### 3.3. Small Bolides

**Figure 13:**
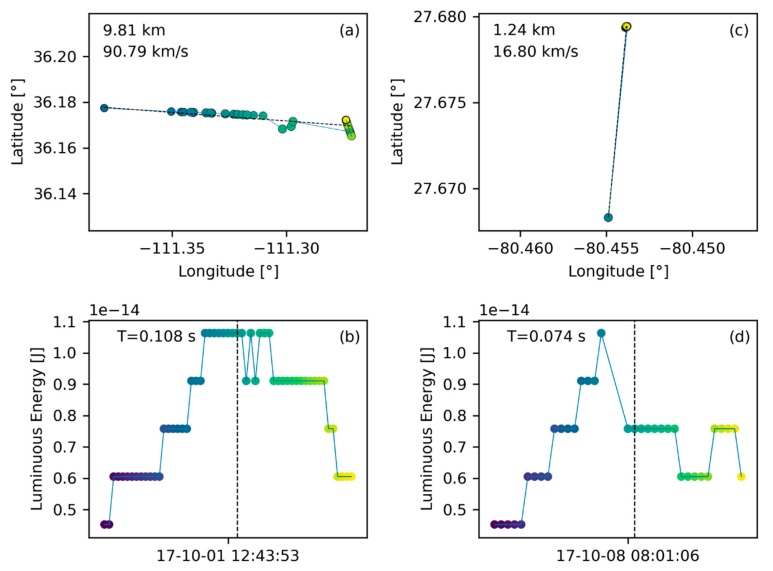
Two small true positive bolide signatures confirmed by All Sky measurements [20]. Groundtrack (**a**) and light curve (**b**) correspond to the same flash. Similarly, (**c**) and (**d**) are one flash.

Small bolide events are particularly interesting because they are more common than large events and will, thus, provide the largest percentage of bolide signatures. Therefore, it is desirable to capture small bolide occurrences. However, extracting small bolides is also more challenging because the noise level in these signatures increases as the GLM instrument reaches its resolution limits. The GLM pixel resolution is 8–14 km and small bolides might not traverse from one pixel to the next while burning up in the atmosphere. This causes challenges for the geolocation algorithm of GLM and yields meteor ground tracks that significantly deviate from line-like behavior. For example, the ground track of a small bolide presented in Figure 13a, features some ground track points that clearly deviate from the line fit. The deviation from a line-like ground track is likely caused by the fact that GLM operates at the lower end of its resolution for small bolide events. 

In addition, for groups that only trigger one pixel of the CCD, GLM tends to place those ground track points at the center of the illuminated pixel producing a cluster of points rather than a line. This behavior is apparent in Figure 13c where two ground point clusters form the start and end of the ground track for that bolide. A similar pattern is visible in Figure 13a. The clusters in that example occur at the beginning and the end because the energy released during those times only triggered a single pixel. Few ground track points are in between the clusters marking the time when the energy releasing bolide passed from one pixel to the other and activating both pixels. Activation of more than one pixel allows the geolocation algorithm of GLM to produce position estimates based on pixel intensity averaging. Even though no connecting points are visible between the clusters in the examples of Figure 13c, it was still possible for the algorithm to detect this bolide and to produce an adequate line fit. 

Unfortunately, this capability breaks down when the bolide does not traverse between pixels and only one pixel is triggered yielding one group cluster. At present, this situation presents a serious challenge to the algorithm which currently leads to the discarding of such signatures. An alternative approach might be necessary that purely focuses on the light curve data and this prospect constitutes future work.

The speed estimates tagged in the top left corner of the ground track plots also suffer from resolution issues for small flashes. For example, the speed estimate for the bolide shown in Figure 13a is unrealistically high. Because of the resolution limitations of GLM it is assumed that the bolide travelled from the center of the first pixel to the second pixel center. However, it is likely that the bolide did not, in fact, travel from the center of one pixel to the next but only occurred on a smaller distance between these two points. The assumption of the longer ground track leads to an overstated speed estimate because the distance covered is longer in the well-known amount of light curve duration. It is important to be cognizant of this artifact behavior when interpreting speed estimates.

The light curve of small bolides presents a challenge for the energy balance filter because the bias towards late peak energy deposition is less pronounced. In fact, the energy balance is calculated to be close to half signature duration in the examples provided in Figure 13. What is more, the received energy barely suffices to trigger the event recording and energy quantization is apparent in the light curves as the energy levels are at the low end of the dynamic range of the CCD (especially visible in Figure 13b,d). Energy quantization might cause poor fit results for the polynomial fit filter reducing the overall bolide score leading to the potential discarding of such signatures. Finally, small bolides contain only a limited number of groups and their signatures, thus, are close to the noisier regime of small group count flashes (see Figure 8). Specifically, the bolide in Figure 13a,b contains 55 and the one in Figure 13c,d only 35 groups. Hence, these two bolides pass the current filter with relatively low scores (0.73 and 0.57 with baseline filter setting) but similar, small bolides might just fall below the threshold. Given that small bolides are frequent and the most numerous source of meteor data, future work should focus on extracting small events more robustly.

### 3.4. General Discoveries

The algorithm design will be a work in progress for some time. As such, it is a challenge to extract representative discovery statistics at this point. Nonetheless, Table 3 lists the algorithm performance in November 2018 and 7 highly likely bolide discoveries have been made in that month.

**Figure 14:**
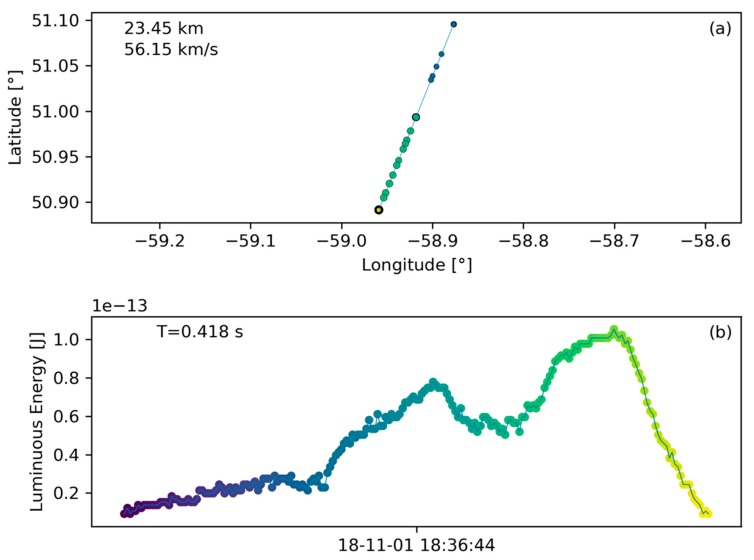
Unconfirmed bolide discovery corresponding to the first entry listed in Table 3. The flash occurred in a remote location over the far eastern shore of Quebec, Canada. Plot (**a**) shows ground track and plot (**b**) shows light curve.

The first three discoveries are unconfirmed (meaning that they have not been observed by other means) and are likely bolides based on their appearance. The other four signatures were observed by other sources such as USG sensors, all-sky cameras or the second GLM instrument on the GOES-17 satellite and could, thus, be confirmed. As an example of an unconfirmed bolide, Figure 14 presents the ground track and light curve information of the first entry listed in Table 3. This data demonstrates that the algorithm is capable of discovering new bolide-like signatures where other systems cannot provide data. This new capability also presents a challenge as the lack of corroborating observations makes confirmations difficult.

### 3.5. Cuban Meteor

**Figure 15:**
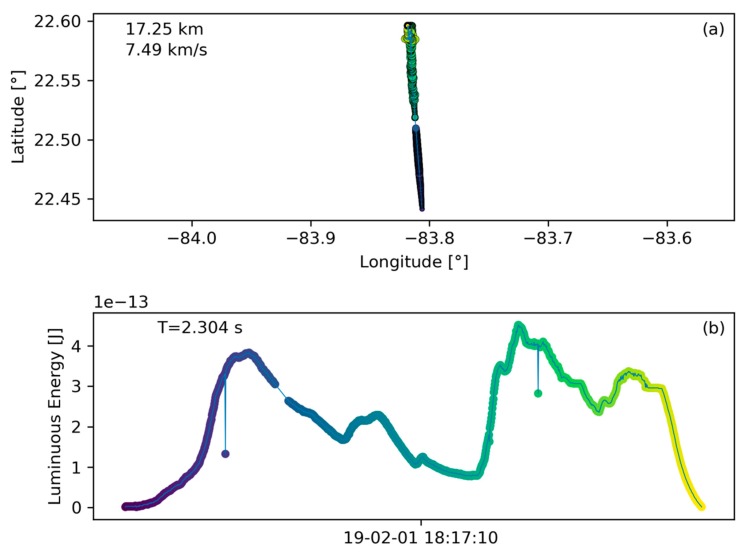
(**a**) Ground track and (**b**) light curve information of the Cuba meteor on 1 February 2019.

The value of GLM as a public, and semi-global coverage meteor detection capability in combination with the automatic signature extraction functionality provided by this algorithm is further exemplified on the meteor that disintegrated over western Cuba on February 1st 2019. Figure 15 presents the ground track and light curve information for that flash. This meteor was widely observed and initial amateur observations collected by the American Meteor Society and through video uploads to the video sharing platform YouTube supported a shallow entry trajectory over Florida towards Cuba [32]. After the authors of this paper were made aware of the meteor, and ran the algorithm, the meteor was immediately recovered in the data. The subsequent publication of the ground track on social media was the first available data that corrected the initial misconception about the entry trajectory [33] that actually only extended few kilometers over Cuba and headed from South to North. It was also the first source of a light curve at the same time. Between the meteor occurrence and the discovery in GLM data, 8.5 hours had passed. It is conceivable that the algorithm can run constantly in the future in which case this time could be reduced significantly. This bolide has also been observed by numerous other sources such as infrasound, radar, and USG sensors.

### 3.6. Data Limitations and Future Work

The GLM instrument was designed to detect lightning in the atmosphere. As such, its assumptions and parameters are selected for that purpose. The geolocation procedure of GLM assumes that the energy emitting event is on top of the cloud cover (assumed to be 6 km at the poles to 14 km at the equator [34]) because this is where lightning emissions occur. However, bolides may occur in altitude bands ranging from the ground to about 100 km and typically they occur higher than the cloud cover level. As such, the geolocation of bolide signatures suffers from parallax effects and will generally be inaccurate depending on the viewing angle from nadir. In the future, it could be possible to constrain the bolide altitude for those discoveries that were detected by both GLM sensors on GOES 16 and GOES 17 enabling stereo vision.

Better understanding of the narrow bandwidth spectral filter is necessary in order to use GLM bolide light curves to calculate total radiant energy. It has been shown by [2] that calculated total optical radiant energies in GLM correspond well to those reported from broad-band USG sensor data which suggests that during the meteoroid’s peak brightness the GLM pass-band is dominated by continuum emission, rather than O I line emission [2]. However, more detailed analysis on how bolide emissions registered by GLM relate to the actually released energy by the bolide is necessary and is currently under way at NASA Ames Research Center. Due to their increased occurrence rate, small bolides are more likely to be seen by multiple observatories. Small bolides are therefore particularly useful to calibrate GLM-based energy estimates with independent observations.

The GLM algorithm that transforms level 0 to the level 2 data product removes knowledge about the accuracy and uncertainty associated, especially, with the bolide light curve. GLM could provide value in the future as a large-quantity source for meteor observations. To increase science value of such an output, the uncertainties associated with GLM measurements will be vital information that is currently not publicly available.

Terrestrial glint is a phenomenon where sunlight is reflected off of calm water surfaces and directly enters the GLM sensor. This high energy input causes unwanted triggering of the GLM CCD and produces a considerable fraction of false positive bolide flashes. This is a known problem that is being addressed by the GLM ground segment. In the future, the addition of a glint filter would be a valuable contribution to this work that will reduce the amount of false positive detections.

Figure 2 shows the coverage area of GLM and it is clear that parts of the Americas are covered by two GLM sensors enabling stereo detections. In the future, this stereo detection of meteors enables a multitude additional benefits such as flight path reconstruction of meteoroids, burst altitude determination, and increased precision in the light curve signal.

The clustering parameters for the collection of groups into flashes (see Table 1) were selected on a trial basis. The authors recommend the usage of the official GLM clustering parameters [1,29] to maintain consistency in the GLM community and it is expected that changing the parameters should not have a detrimental effect on bolide detection.

As the bolide extraction algorithm reaches maturity and enters routine operations, reliable statistics on its extraction efficiency will be gathered. This data can be used in the future to quantify the algorithm’s efficiency by comparing the detection rate to the statistical meteor flux. 

The GLM extraction algorithm presented here is a first step and leaves room for improvement. Improvements can be realized through further modification and tuning of the filters. However, as large quantities of data are produced by GLM, machine learning approaches appear to be a promising application to process and classify GLM data. An algorithm such as presented in this paper can be a useful first step to extract and classify training data for such an approach. 

## 4. Conclusions

The GLM instrument on NOAA GOES 16 and 17 satellites is originally intended to record lightning events. Here a new algorithm has been presented that utilizes publicly available L2 data from the GLM instrument to recover meteor signatures in the GLM data product. We highlight the signature differences between lightning and bolide ground tracks as well as light curves. These differences are exploited by filter functions to identify bolide signatures. The algorithm architecture and the derivation of the individual filters is presented. The algorithm discards 98.45% of GLM produced files in its baseline setting, thus, condensing the data effectively to the remaining 1.55% of the original data that include bolide-like signatures. Each filter’s performance was assessed and the filter baseline setting is shown to yield a practical parameter combination that preserves the 6 known true positive bolide signatures (for example, Figure 6 and Figure 13) while discarding the vast majority of generic GLM L2 files. Several bolides have been identified in L2 data using the algorithm and these signatures have been confirmed as real bolides with US government data or with ground based camera systems. Several more unconfirmed discoveries have been made of which three are listed in Table 3 and one is presented in Figure 14. The case of the Cuban meteor of Feb 1st 2019, for which the algorithm in combination with GLM could quickly provide the first light curve and correct ground track, demonstrated nicely the new capability. This outcome demonstrates the value of GLM in combination with the automatic bolide algorithm as a source of meteor measurements drawing on semi-global coverage and providing publicly available data of meteor ground tracks and light curves. The key contribution of this algorithm is that it extracts bolide-like signatures from the vast amount of GLM data and therefore makes the GLM product manageable for bolide discoveries. It is a first step and it is a virtual certainty that more unflagged bolide signatures, especially smaller ones, are present in the data. Small bolide flashes remain a challenge as the signatures reside at the resolution limit of the GLM instrument. Future work should build on this contribution and increase discovery efficiency by tuning and modifying filters, as well as through alternative approaches such as machine learning. Additional work needs to focus on the processing and qualification of GLM derived bolide data to increase utility for scientific purposes. In summary, it has been demonstrated that this algorithm serves its intended purpose and can be used to extract bolide signatures from GLM data.

## Figures and Tables

**Table 1:** Spatial and temporal threshold values to meet closeness requirement collecting groups into flashes.

Closeness Aspect	Value
Spatial separation in longitude	≤0.05°
Spatial separation in latitude	≤0.05°
Temporal separation	≤0.2 s

**Table 2:** Filter parameter set as they apply to the sigmoid function of the filters.

Filter	Parameter a	Parameter b
**Group Count**	0.07	25
**Line Fit**	3	−5
**Energy Balance**	25	0.3
**Max Group to Line Distance**	80	0.4
**Polynomial Fit**	3	−2
**Duration**	2	6

**Table 3:** Bolide extraction algorithm performance in November 2018. If more than one observation data entry is shown, the bolide can be considered confirmed. The entry “all-sky” in the Observation Data column refers to ground based camera observations of that bolide.

Date (YY/MM/DD) Time (UT)	Latitude (degree)	Longitude (degree)	Duration (s)	Lightcurve Energy (J)	Observation Data
18/11/1 18:36:44	51.0N	58.9W	0.462	1.05E-11	GLM-16
18/11/3 12:36:21	5.0N	102.3W	0.155	1.49E-12	GLM-16
18/11/11 7:58:29	34.1N	35.6W	0.078	7.25E-13	GLM-16
18/11/12 4:58:15	29.1N	85.9W	0.837	2.09E-12	GLM-16, all-sky
18/11/15 8:02:44	42.4N	52.8W	0.877	1.33E-10	USG, GLM-16
18/11/20 12:17:52	34.9N	118.4W	0.36	9.55E-12	GLM-16, GLM-17, all-sky
18/11/22 13:10:46	33.1N	122.2W	0.324	1.14E-11	GLM-16, GLM-17, all-sky
